# Use of Human Intravenous Immunoglobulin Therapy in Two Dogs with Non-Neoplastic Bone Marrow Disorders Refractory to Immunosuppressive Therapy

**DOI:** 10.3390/vetsci13020145

**Published:** 2026-02-03

**Authors:** Eun-Ji Kim, Hyun-Jung Han

**Affiliations:** 1 Department of Veterinary Emergency and Critical Care, College of Veterinary Medicine, Konkuk University, Seoul 05029, Republic of Korea; eunji8087@konkuk.ac.kr; 2 KU Center for Animal Blood Medical Science, Konkuk University, Seoul 05029, Republic of Korea

**Keywords:** precursor-targeted immune-mediated anemia, myelodysplastic syndrome, regenerative response, transfusion term, intravenous immunoglobulin

## Abstract

Immune-mediated non-neoplastic bone marrow disorders (NNBMD), including precursor-targeted immune-mediated anemia (PIMA) and myelodysplastic syndrome (MDS), can cause non-regenerative anemia in dogs and often fail to respond to immunosuppressive treatment. Human intravenous immunoglobulin (hIVIG) has been proposed as an immunomodulatory therapy for refractory hematologic disease in humans, but evidence in veterinary patients remains limited. This case report describes the adjunctive use of hIVIG and the associated clinical responses observed in two dogs with refractory NNBMD. A 10-year-old (3.2 kg) spayed female Maltese (case 1) showed persistent non-regenerative anemia, with hematocrit (HCT) 15.6–20.2% and reticulocyte production index (RPI) 0.12–0.69, requiring four transfusions over 10 months, and was diagnosed with PIMA based on bone marrow cytology demonstrating destruction of erythroid precursors. A 7-year-old (9.3 kg) intact female Dachshund (case 2) had sustained non-regenerative anemia (HCT 10.8–20.9%, RPI 0.12–0.40) necessitating eight transfusions over 20 months and was diagnosed with MDS characterized by dyserythropoiesis. Human intravenous immunoglobulin (0.5 g/kg over 6 h) was administered six times in case 1 and seven times in case 2 over a four-month period. After initiating hIVIG therapy, RPI increased (0→1.80 and 0.25→2.10 in cases 1 and 2), and HCT remained above 20% without further transfusions. To our knowledge, this is the first clinical report demonstrating the adjunctive use of hIVIG and associated hematologic improvement in canine NNBMD.

## Simple Summary

Immune-mediated non-neoplastic bone marrow disorders in dogs are uncommon conditions characterized by impaired erythropoiesis and resultant non-regenerative anemia. Affected dogs develop severe anemia and require repeated blood transfusions, and they may not improve with commonly used immunosuppressive medications. Human intravenous immunoglobulin, a concentrated preparation of antibodies collected from healthy people, has been used in human medicine to regulate the immune system in refractory immune disorders. This case report describes two dogs with chronic, non-regenerative anemia associated with non-neoplastic bone marrow disorders that did not improve despite multiple transfusions and immunosuppressive treatment. Both dogs received several doses of human intravenous immunoglobulin over four months. After treatment, erythroid regeneration improved, with the reticulocyte production index increasing from 0.42 to 3.81 in case 1 and from 0.27 to 1.34 in case 2. The average transfusion interval was prolonged (10.3 to 20.9 weeks in case 1; 11.6 to 31.7 weeks in case 2), and the duration of maintaining a normal hematocrit increased. These findings suggest that human intravenous immunoglobulin may represent adjunctive therapy for dogs with non-neoplastic bone marrow disorders refractory to standard immunosuppressive treatment, improving hematologic parameters and reducing transfusion requirements.

## 1. Introduction

Bone marrow disorders are primary causes of non-regenerative anemia in dogs, classified as neoplastic, hypoplastic, or dysplastic [1]. Non-neoplastic bone marrow disorders (NNBMD), including non-regenerative immune-mediated anemia (NRIMA), precursor-targeted immune-mediated anemia (PIMA), myelodysplastic syndrome (MDS), myelofibrosis, and essential thrombocytosis, result from impaired maturation or destruction of erythroid precursors in the marrow, leading to ineffective erythropoiesis and persistent non-regenerative anemia [1,2,3].

Treatment for NNBMD typically involves immunosuppressive therapies, such as prednisolone, cyclosporine, and azathioprine [3,4]. Prognosis depends on treatment response and complications associated with severe anemia or immunosuppressive therapy [5]. In PIMA, regenerative responses are generally observed within 30–60 days; however, 17–33% of dogs fail to respond to immunosuppressive therapy and may require repeated transfusions [5,6]. In dogs, MDS is a rare and poorly characterized form of NNBMD. Most available information is derived from small case series and isolated case reports [1,3]. In the largest retrospective study to date, primary MDS was identified in 12 of 220 canine bone marrow specimens and was further classified into refractory anemia and myelodysplasia with excess blasts, which differed markedly in clinical behavior and prognosis [1]. Dogs with refractory anemia often exhibited prolonged survival and occasional response to supportive or erythropoietin-based therapy, whereas dogs with myelodysplasia, with increased myeloblasts, generally had poor outcomes and short survival times, with limited response to conventional treatments [1]. Despite these limited reports, population-level data remain scarce, standardized diagnostic criteria are inconsistently applied, and no evidence-based treatment guidelines have been established in veterinary medicine. In cases unresponsive to immunosuppressive therapy, alternative treatment options are limited.

In human medicine, human intravenous immunoglobulin (hIVIG) has been widely used as an immunomodulatory therapy across a broad range of immune-mediated and inflammatory conditions and applied as an adjunctive therapy in selected refractory hematologic disorders [7,8]. Reported benefits include immunomodulation through Fc-receptor blockade, cytokine modulation, and suppression of autoreactive immune responses, with particular utility in patients who are refractory to or intolerant of conventional immunosuppressive therapies [7,8]. However, responses are varied and often incomplete, and their use is generally supported by limited evidence rather than standardized treatment guidelines [5,7]. In veterinary medicine, hIVIG has been most commonly documented for immune-mediated diseases such as immune-mediated thrombocytopenia, immune-mediated hemolytic anemia, Evans syndrome, and selected autoimmune neurologic or dermatologic conditions, where it may provide transient or adjunctive benefit [7,8]. Nevertheless, evidence supporting its use for NNBMD, including PIMA and MDS, remains extremely limited.

This report describes two dogs with refractory non-regenerative anemia due to NNBMD (PIMA and MDS) that were unresponsive to conventional immunosuppressive therapy. To our knowledge, this is the first clinical report documenting the adjunctive use of hIVIG in dogs with NNBMD.

## 2. Case Presentation

### 2.1. Case 1

A 10-year-old spayed female Maltese dog (3.2 kg) presented to our institution’s Veterinary Medical Teaching Hospital (VMTH) after being referred for recurrent syncope, anemia, and thrombocytopenia. The dog had a history of pulmonary hypertension, diarrhea, and a hypercoagulable state without clinical evidence of bleeding diathesis, and was on medications, including immunosuppressants (cyclosporine, 6 mg/kg q 24 h), pulmonary vasodilator therapy (sildenafil, 1.5 mg/kg q 12 h), gastroprotectants (omeprazole, 1 mg/kg q 12 h), and antiplatelet agent (clopidogrel, 1 mg/kg q 24 h).

The underlying causes of the gastrointestinal signs and suspected hypercoagulable state were not definitively determined prior to referral but were considered potentially multifactorial, possibly related to prolonged immunosuppressive therapy and underlying cardiopulmonary disease. Cyclosporine dosing had been adjusted at the referring local hospital based on therapeutic drug monitoring prior to referral. Sildenafil had been initiated at a referring local hospital based on echocardiographic evidence of moderate pulmonary hypertension, including moderate tricuspid regurgitation (TR velocity 3.86 m/s; estimated tricuspid regurgitation pressure gradient (TRPG), 59.6 mmHg). On follow-up echocardiography performed approximately six months later, the estimated TRPG had increased to 65 mmHg and was accompanied by worsening clinical signs, prompting escalation of the sildenafil dose to 1.5 mg/kg q 12 h.

Over the 12 months preceding referral, the dog had been treated with immunosuppressive drugs, including prednisolone, mycophenolate mofetil, cyclosporine, and leflunomide, without evidence of sustained hematologic improvement. Detailed information regarding the dosing, treatment durations, and reported hematologic responses for each immunosuppressive drug is provided in Supplementary Table S1. The reticulocyte production index (RPI) (median: 0.14, range: 0.04–0.24) consistently indicated non-regenerative anemia. The dog received four blood transfusions over 10 months at a local hospital, with the intervals between each transfusion progressively shortened to 6 months, 54 days, 38 days, and 30 days. Each transfusion resulted in a temporary increase in hematocrit (HCT). Additionally, from the third transfusion onwards, persistent incompatibility was observed in the cross-match test. The dog was referred for further evaluation and blood transfusion.

On presentation, physical examination revealed a body temperature of 39.0 °C, a heart rate of 144 beats/min, and a respiratory rate of 42 breaths/min. The gingival mucous membranes were pale pink, and the capillary refill time was normal at 1.5 s. A complete blood count revealed macrocytic hypochromic anemia with an HCT of 17.5% (reference interval [RI]: 37.3–61.7%), hemoglobin concentration of 5.5 g/dL (RI: 13.1–20.5 g/dL), mean corpuscular volume (MCV) of 74.2 fL (RI: 61.6–73.5 fL), and mean corpuscular hemoglobin concentration (MCHC) of 31.4 g/dL (RI: 32–37.9 g/dL). Both white blood cell (WBC) (4.82 × 10^9^/L [RI: 5.05–16.76 × 10^9^/L]) and platelet (122 K/µL [RI: 148–484 K/µL]) counts were mildly decreased. However, mild leukopenia and thrombocytopenia were present at initial evaluation and persisted at similar levels during early follow-up without progressive worsening. These abnormalities showed no temporal association with clinical deterioration and were therefore not considered primary contributors to disease progression. Subsequent complete blood count revealed normalization of leukocyte and platelet counts (WBC, 7.6 × 10^9^/L; platelet count, 206 K/µL), and no recurrent leukopenia or thrombocytopenia was observed on follow-up evaluations. Although the reticulocyte count was 53.8 K/µL (RI: 10–110 K/µL), which is higher than typically observed in erythroid aplasia, the RPI remained low (0.59), indicating an inadequate and ineffective erythroid regenerative response. Blood smear examination showed no evidence of hemolysis, and the autoagglutination test was negative. Serum chemistry tests revealed elevated C-reactive protein (CRP) levels (2.1 mg/dL [RI: 0.1–1 mg/dL]). The D-dimer concentration was elevated at 351.54 ng/mL [RI: 50–250 ng/mL], and the prothrombin time and activated partial thromboplastin time were normal (13 s [RI: 11–17 s] and 96 s [RI: 72–102 s], respectively). Thromboelastography analysis indicated hypercoagulability with a normal reaction time, shortened clotting time, and increased maximum amplitude and angle. Serum cobalamin and folate concentrations had been previously evaluated at the referring hospital and were within reference intervals; therefore, vitamin B12 or folate supplementation was not initiated. Blood typing using a commercial immunochromatographic test (Lab test QuickTest dog erythrocyte antigen [DEA] 1, Alvedia, Limonest, France) and an agglutination card test (RapidVet-H Canine DEA 4 Agglutination Card Test, RapidVet-H Canine DAL Agglutination Card Test, DMS, Flemington, NJ, USA), revealed DEA 1-positive, DEA 4-positive, and DAL-positive blood types.

Real-time polymerase chain reaction (PCR) results for the canine anemia pathogen test confirmed negativity for 10 pathogens (*Anaplasma, Ehrlichia*, *Babesia* spp., *Leptospira* spp., *Bartonella* spp., *Hemotropic mycoplasma*, *Rickettsia* spp., *Lyme*, *Hepatozoon* spp., and *Theileria* spp.). PCR for antigen receptor rearrangement showed no clonal rearrangements in immunoglobulin or T-cell receptor genes, excluding lymphoma and leukemia. Bone marrow aspiration under general anesthesia revealed cytological findings with a myeloid-to-erythroid (M/E) ratio of 0.4:1 (RI 0.75–3:1), indicating erythroid hyperplasia. The predominance of rubricytes and metarubricytes suggested immune-mediated destruction of erythrocyte precursors following the metarubricyte stage. Based on these findings and diagnostic criteria from a previous study [4,9] (persistent non-regenerative anemia with HCT ≤ 30%, reticulocyte count < 76,000 reticulocytes/µL, ineffective bone marrow erythropoiesis, and clinical signs), the dog was diagnosed with PIMA.

For the fifth transfusion (the first performed at our institution), multiple major cross-match tests were performed using an immunochromatographic cross-matching kit (LabTest xm, Alvedia, Lyon, France). Due to suspected alloantibody formation, compatibility was identified only after the ninth test, with donor blood typed as DEA 1-, DEA 4-, and DAL-positive. A leukoreduced packed red blood cell unit (DEA 1-positive) was transfused, increasing the hematocrit from 17.5% to 37.2%, without any evidence of hemolysis or transfusion reaction.

After the fifth blood transfusion, which increased the hematocrit from 17.5% to 37.0%, hIVIG therapy was initiated and subsequently administered six times over a 4-month period. The intervals between hIVIG administrations were determined based on the patient’s clinical progression. The second dose was administered 3 weeks after the initial treatment, the third dose 2 weeks later, and the fourth and fifth doses at 1-week intervals, resulting in a total of five administrations by treatment week 8. After the fifth administration, the dog returned to treatment week 9 for routine monitoring. However, because the dog remained clinically stable, the owner did not return thereafter, resulting in a 10-week interruption in therapy. The dog re-presented at treatment week 19 with recurrent non-regenerative anemia, and the sixth hIVIG dose was administered 11 weeks after the fifth dose.

**Figure 1:**
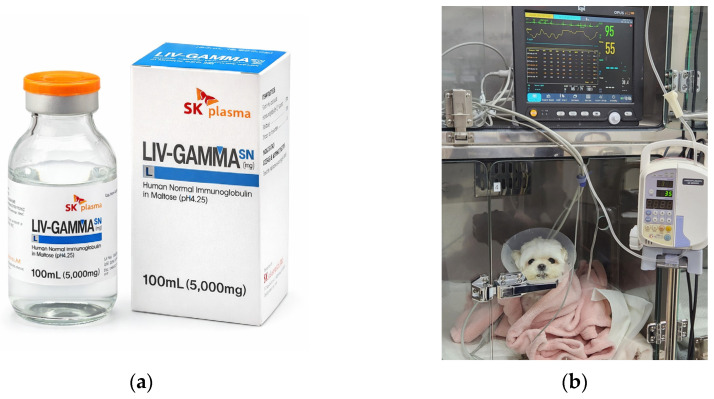
Administration of human intravenous immunoglobulin (hIVIG) in case 1. (**a**) Commercial human intravenous immunoglobulin (hIVIG; LIV-GAMMA SN 5%, SK plasma, Korea) used for treatment. (**b**) Clinical monitoring of the patient during hIVIG infusion. The dog received 0.5 g/kg of hIVIG over 6 h via a constant rate infusion (CRI) using an infusion pump, with continuous ECG and blood pressure monitoring in the intensive care unit.

As premedication, chlorpheniramine (0.2 mg/kg) was administered subcutaneously. The hIVIG was then administered at a dose of 0.5 g/kg over 6 h, followed by a constant rate infusion (CRI) [7], initiating at a slow rate (0.1 mL/kg/min) and gradually increasing every 30–60 min to a maintenance rate not exceeding 0.8 mL/kg/min (Figure 1) [8]. Detailed information is provided for hIVIG therapy, which was administered at our institution using a response-driven protocol, whereas other treatments consisted of standard therapies initiated prior to referral or without measurable clinical benefit.

**Figure 2:**
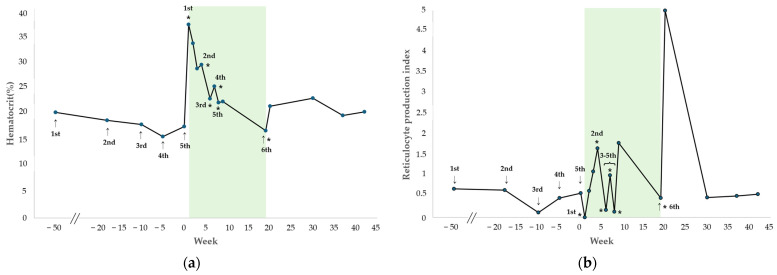
Changes in hematocrit (HCT) and reticulocyte production index (RPI) after hIVIG in case 1. Changes in HCT (**a**) and RPI (**b**) after receiving blood transfusions (arrows) and hIVIG (asterisks) in case 1. The colored boxes indicate the periods of hIVIG administration.

The changes in HCT and RPI after each hIVIG administration are shown in Figure 2. During the regular administration period up to the fifth dose (treatment weeks 1–8), both parameters remained stable (mean HCT: 24.3%, range 22.1–29.3%; mean RPI: 0.96, range 0.14–1.80), and the dog showed no clinical signs of anemia and did not require any additional blood transfusions.

Three weeks after initiating the first hIVIG treatment, the second administration resulted in HCT of 29.3%, reticulocyte count of 78.6 K/µL, and RPI of 1.67, indicating a regenerative response. At this time, tapering of cyclosporine was initiated owing to worsening gastrointestinal signs, including persistent diarrhea, an elevated CRP of 20.2 mg/dL (RI 0.1–1 mg/dL), and diffuse thickening of the descending colon wall on abdominal ultrasonography. The dog had been receiving cyclosporine at presentation to our VMTH, as prednisolone, mycophenolate mofetil, and leflunomide, previously prescribed at a local hospital, had already been discontinued due to insufficient therapeutic response. After the fifth blood transfusion performed at our institution, the dog received hIVIG at regular intervals. During this period, the HCT demonstrated a gradual decline but remained above 22% for 8 consecutive weeks, and no clinical signs of anemia were observed, without requiring further transfusion. Regarding the RPI, non-regenerative responses (RPI < 0.5) were identified at treatment weeks 6 and 8 during the period of hIVIG administration. However, in all cases, the RPI increased to at least 1.0 within one week after hIVIG dosage, indicating a regenerative response. Subsequently, during a follow-up phone call, the owner reported that the dog remained clinically stable and did not return for further evaluation after treatment week 9, which occurred one week after the fifth hIVIG administration. Detailed transfusion and laboratory data are provided in Supplementary Table S2.

Eleven weeks after the fifth hIVIG administration (treatment week 19), the dog developed recurrent anemia with clinical signs, including lethargy and pale mucous membranes, and required another blood transfusion at the VMTH. The pre-transfusion complete blood count revealed the following values: HCT = 16.7%; hemoglobin concentration = 5.4 g/dL, MCV = 73.2 fL, MCHC = 32.3 g/dL, reticulocyte count = 26 K/µL, RPI = 0.47, WBC = 4.04 × 10^9^/L, and PLT = 138 K/µL. The post-transfusion HCT was 25.2%. The following day, the sixth hIVIG was administered. One week after the sixth hIVIG administration, HCT improved to 21.4%, with a reticulocyte count of 145.7 K/µL and RPI of 5, indicating a regenerative response. After the sixth hIVIG administration, the patient was monitored for 23 weeks, during which the HCT remained stable with a mean of 24.5% (range, 21.4–33.4%) and the RPI was maintained at a mean of 3.81 (0.14–5.00). Throughout this period, the dog exhibited no clinical signs of anemia. Patient characteristics and comparisons of regenerative indicators pre- and post-HIVIG administration for case 1 are shown in Table 1.

### 2.2. Case 2

A 7-year-old, intact female dachshund dog (9.3 kg) presented to a VMTH with non-regenerative anemia. There was a history of gastrointestinal signs and a hypercoagulable (prothrombotic) state during immunosuppressive therapy prior to presentation. For 20 months prior to referral, the dog had been treated with multiple immunosuppressive drugs, including prednisolone (1 mg/kg q 12 h), mycophenolate mofetil (10 mg/kg q 12 h), cyclosporine (up to 6 mg/kg q 24 h, with dose adjustments based on therapeutic drug monitoring), and leflunomide (3 mg/kg q 24 h), either sequentially or in combination, without documented sustained hematologic improvement. Despite these treatments, no sustained increase in hematocrit or reticulocyte production was reported prior to referral. Detailed information regarding the dosing, treatment durations, and reported hematologic responses for each immunosuppressive drug is provided in Supplementary Table S1. Before referral, the dog had been treated with gastrointestinal protectants (omeprazole: 1 mg/kg q 12 h) and an antiplatelet agent (clopidogrel: 2 mg/kg q 24 h), initiated due to concern for thrombotic risk. The dog received eight blood transfusions over 20 months at a referring hospital. On average, the dog received transfusions every 2–3 months. Temporary increases in HCT were observed after all eight transfusions; however, RPI (median: 0.61, range: 0.22–0.90) consistently indicated non-regenerative anemia.

On presentation, physical examination revealed a body temperature of 39.2 °C, a heart rate of 142 beats/min, and a respiratory rate of 48 breaths/min. The gingival mucous membranes were pale pink, and the capillary refill time was normal (1.5 s). A complete blood count revealed macrocytic hypochromic anemia (HCT = 13.3%, hemoglobin concentration = 5.0 g/dL, MCV = 70.8 fL, and MCHC = 30 g/dL). Elevated WBC (30.89 × 10^9^/L) and platelet (641 K/µL) counts were observed. The reticulocyte count was 47.9 K/µL (RI: 10–110 K/µL), with an RPI of 0.25, indicating non-regenerative anemia. Blood smear examination showed no evidence of hemolysis, and the autoagglutination test was negative. Serum chemistry tests showed increased lactate (7.49 mmol/L), triglyceride (273 mg/dL), aspartate aminotransferase (51 U/L), and CRP (1.9 mg/dL) levels. The D-dimer concentration was 127.72 ng/mL [RI: 50–250 ng/mL], prothrombin time was 15 s [RI 11–17 s], and activated partial thromboplastin time was 82 s [RI: 72–102 s]. Thromboelastography analysis indicated hypercoagulability with a normal reaction time, clotting time, and angle, along with an increased maximum amplitude. Blood typing using a commercial immunochromatographic test (Lab test QuickTest dog erythrocyte antigen [DEA] 1, Alvedia, Limonest, France) and an agglutination card test (RapidVet-H Canine DEA 4 Agglutination Card Test, RapidVet-H Canine DAL Agglutination Card Test, DMS, Flemington, NJ, USA) revealed DEA 1-positive, DEA 4-positive, and DAL-negative blood types.

Thyroid function test revealed low total-T4 (0.8 µg/dL [RI: 1.0–4.0 µg/dL]), low free-T4 (0.5 ng/dL [RI: 0.6–3.7 ng/dL]), and high thyroid-stimulating hormone (0.72 ng/mL [RI: 0.05–0.42 ng/mL]). Treatment for hypothyroidism was initiated with levothyroxine (0.01 mg/kg PO q 12 h). A canine anemia pathogen test using real-time PCR showed negative results for 10 species, as in case 1, and antigen receptor rearrangement PCR showed no specific findings. Abdominal ultrasonography was performed to evaluate potential secondary causes of non-regenerative anemia. No evidence of intra-abdominal hemorrhage, neoplasia, or infiltrative disease was identified. The reproductive system was unremarkable, with both ovaries and uterine horns within normal limits, making estrogen-producing ovarian pathology and hyperestrogenism unlikely. No imaging findings suggestive of secondary bone marrow suppression were identified.

Bone marrow aspiration from the humerus revealed no evidence of an immune-mediated process targeting erythroid precursors or overt neoplastic processes. However, atypical features, such as binucleation observed in some erythroid cells, suggest dyserythropoiesis, indicating a problem during red blood cell maturation. Based on these findings, the dog was diagnosed with MDS. However, due to low cellularity, the M/E ratio was unmeasurable, and the limited number of abnormal cells made it challenging to specify the MDS subtype.

For the ninth blood transfusion, which represented the dog’s ninth overall but the first performed at our institution, multiple major cross-match tests with a commercial immunochromatographic cross-match test kit (LabTest xm; Alvedia, Lyon, France) showed incompatibility. Therefore, a standard laboratory cross-match test (manual major cross-match) was performed using blood from 10 donors to identify the blood with the lowest agglutination scale for transfusion [10]. The dog received a DEA-1 positive leukoreduced packed red blood cell transfusion, resulting in a post-transfusion HCT of 36.4% without any transfusion reactions.

The first hIVIG administration was performed immediately prior to the ninth blood transfusion, and a total of seven hIVIG administrations were performed over a 4-month period. During treatment week 0, the second and third doses were administered on consecutive days within 24 h of the first dose, following the same protocol used in case 1. This initial closely spaced dosing was empirically selected to provide short-term immunomodulatory support in the setting of severe, transfusion-dependent anemia, rather than as part of a predefined or standardized treatment protocol. Subsequent hIVIG administrations were not performed at fixed intervals but were determined on a response-driven basis, guided by serial hematologic monitoring and clinical status. Specifically, repeat dosing was considered when hematocrit values declined toward the transfusion threshold (<20%), the RPI remained persistently low (<1.0), and/or clinical signs consistent with anemia recurred. Based on these criteria, the fourth, fifth, and sixth doses were administered at treatment weeks 1, 3, and 4, respectively, resulting in a total of six administrations by treatment week 4. These dosing decisions were intended to provide supportive management and delay the need for additional transfusions, rather than to induce definitive disease remission. After the sixth administration, the dog showed clinical improvement and did not re-present for 11 weeks. The dog returned at treatment week 15 for routine monitoring, and because no clinical signs of anemia were observed, further hIVIG was not administered. The dog re-presented at treatment week 18 with recurrent non-regenerative anemia, for which a seventh hIVIG dose was administered. Subsequently, hIVIG was administered using the same dosage and protocol as in case 1.

**Figure 3:**
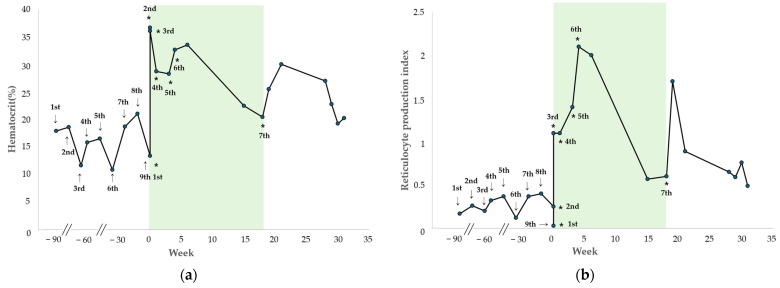
Changes in hematocrit (HCT) and reticulocyte production index (RPI) after hIVIG in case 2. Changes in HCT (**a**) and RPI (**b**) after receiving blood transfusions (arrows) and hIVIG (asterisks) in case 2. The colored boxes indicate the periods of hIVIG administration.

The changes in HCT and RPI after each hIVIG administration are shown in Figure 3. During the initial period of regular hIVIG administration up to the sixth dose, the HCT progressively improved and remained with a mean of 32.2% (range, 28.1–36.4%), and the RPI remained at a mean of 1.33 (range, 0.03–2.10). Throughout this period, the dog showed no clinical signs of anemia and did not require additional blood transfusions.

Two days after initiation of hIVIG treatment, at the third administration, the HCT was 35.8%, with a reticulocyte count of 171.4 K/µL and RPI of 1.10, indicating a regenerative response. One week later, at the fourth administration, the HCT was 28.5%, the reticulocyte count was 66 K/µL, and the RPI remained 1.10, consistent with a regenerative response. The fifth treatment was administered 21 days after the treatment initiation, at which time the HCT was 28.1%, the reticulocyte count was 158.4 K/µL, and the RPI was 1.4. At that time, tapering of prednisolone and mycophenolate mofetil was initiated because of elevated pancreatic enzyme levels (>1000 ng/mL [RI: 200–400 ng/mL]) and recurrent gastrointestinal symptoms, such as diarrhea and hematochezia. One week later, during the sixth hIVIG administration, the HCT was 32.4%, the reticulocyte count was 163.4 K/µL, and the RPI was 2.1, indicating a regenerative response. After two additional weeks (treatment week 6), during monitoring, the HCT was maintained at 33.3%, the reticulocyte count was 231.8 K/µL, and the RPI was 2.0 with persistent regeneration. Subsequently, during a follow-up phone call, the owner reported that the dog remained clinically stable after the sixth hIVIG administration and did not return for re-evaluation for 9 weeks. The dog was presented at treatment week 15 for routine monitoring, at which time the HCT was 22.3%, and the RPI was 0.57. No clinical signs of anemia were observed, and because the owner declined additional treatment, another hIVIG dose was not administered. Detailed transfusion and laboratory data are provided in Supplementary Table S3.

Fourteen weeks after the sixth administration (treatment week 18), the dog presented to the VMTH with lethargy. A complete blood count revealed an HCT of 20.3%, a reticulocyte count of 66.2 K/µL, and an RPI of 0.6, consistent with recurrent non-regenerative anemia, and the seventh hIVIG dose was administered. One week after the seventh administration, the HCT increased to 25.3%, with a reticulocyte count of 222.2 K/µL, and an RPI of 1.7, indicating a regenerative response. Thereafter, the dog was monitored for 13 weeks, during which the HCT remained stable with a mean of 24.0% (range, 19.1–29.8%), and the RPI was maintained at a mean of 0.85 (range, 0.49–1.70). As in case 1, no clinical signs of anemia were observed after the final administration, without requiring further blood transfusions. Patient characteristics and comparisons of regenerative indicators pre- and post-HIVIG administration for cases 1 and 2 are shown in Table 1.

## 3. Discussion

NNBMDs, such as PIMA and MDS, are rare in dogs, with the incidence rate of associated anemia unknown. PIMA, a type of NRIMA, involves ineffective erythropoiesis from immune-mediated targeting of erythroid precursors [5]. Prognostic factors for NRIMA include the corrected reticulocyte percentage, severity of reticulocytopenia, and thromboembolic events. Dogs with PIMA have a median survival time of approximately 2.5 years, but this drops significantly to 54 days in cases with thromboembolic events [5]. Given the association between PIMA and thromboembolic complications, antiplatelet therapy was administered in case 1 due to suspected hypercoagulability and concurrent pulmonary hypertension. This intervention was intended for thrombotic risk mitigation and was not considered to influence the interpretation of hematologic response to hIVIG. Dysmyelopoiesis, another NNBMD, is a hematologic disorder characterized by dysplasia and other morphological abnormalities in blood or bone marrow cells and includes MDS, congenital dysmyelopoiesis, and secondary dysmyelopoiesis [1,11]. The cause of primary MDS remains unclear; however, it may involve genetic mutations in hematopoietic stem cells, and survival is generally short due to potential progression to acute myeloid leukemia [11,12,13]. Diagnosis of NNBMDs is established by integrating persistent non-regenerative anemia with bone marrow cytology or histopathology demonstrating ineffective erythropoiesis, dysplastic changes, or immune-mediated destruction of erythroid precursors [1,2,3,5].

In case 1, the dog had received four blood transfusions prior to referral and exhibited persistent refractory non-regenerative anemia (hematocrit 17.5%, reticulocyte count 53,800/µL, RPI 0.59). The diagnosis of PIMA was based on bone marrow findings demonstrating ineffective erythropoiesis with marked erythroid hyperplasia and immune-mediated destruction of erythroid precursors beyond the metarubricyte stage, consistent with the diagnostic criteria proposed by Assenmacher et al. [5]. Although mild leukopenia and thrombocytopenia were noted at the initial evaluation, these abnormalities were transient, non-progressive, and resolved spontaneously to within reference intervals on follow-up (WBC, 7.6 × 10^9^/L; platelet count, 206 K/µL). Importantly, the lack of progression and subsequent normalization of these cell lines indicated that these findings were not consistent with true pancytopenia or global bone marrow failure. Rather, the hematologic profile supported a selective, precursor-targeted erythroid disorder, thereby strengthening the diagnosis of PIMA and arguing against alternative diagnoses such as aplastic processes. In case 2, the dog received eight blood transfusions before referral with refractory anemia (HCT 13.3%, reticulocyte count 47,900/µL, RPI 0.25). MDS was confirmed by bone marrow cytology with dyserythropoiesis, meeting diagnostic criteria with non-regenerative anemia and dysplastic changes, including binucleation in erythroid cells [1].

For canine NNBMDs, including PIMA and MDS, immunosuppressive therapy is considered the principal therapeutic option. PIMA is primarily treated with glucocorticoids, with agents like cyclosporine or mycophenolate mofetil added if needed [6,8,14]. In a prior study, 83% of dogs diagnosed with PIMA and treated with immunosuppressive therapy developed a regenerative response, with a median time to response of 29 days [5]. Immunosuppressive therapy is recommended for at least 60 days to differentiate between responders and non-responders, even if no response is observed by 30 days [6]. For MDS, although no formal guidelines exist, recombinant human erythropoietin and prednisone are potential treatments, along with blood transfusions [4]. In our cases, anemia persisted without a regenerative response despite over 60 days of various immunosuppressive drugs, necessitating consideration of new treatment options [6].

Alternative treatment options for refractory NNBMD in dogs are limited and have been reported only rarely. In 2023, oclacitinib, a Janus kinase-1 inhibitor, was administered in combination with conventional immunosuppressive therapy in two dogs with PIMA that failed to achieve remission, resulting in partial clinical improvement [14]. Similarly, a 2015 case report described the use of aclarubicin as a differentiation-inducing agent in a dog with MDS, in which erythroid maturation was partially restored [15]. However, both reports were single-case descriptions, and no treatment has been validated in larger populations, leaving no established alternative therapy for refractory NNBMD in veterinary medicine [14,15].

In human medicine, several therapeutic strategies beyond immunosuppression, such as IVIG, hypomethylating agents (azacitidine and decitabine), cytokine-based therapies, and chemotherapy, have been investigated for bone marrow failure syndromes, including MDS [1,11]. Nevertheless, none of these modalities have consistently demonstrated durable efficacy across large patient populations, and no universally accepted alternative to immunosuppression exists. Among these therapies, IVIG has shown clinical benefit in subsets of patients with MDS and immune-mediated marrow failure [16]. In human MDS, its therapeutic effects are thought to be mediated through anti-idiotype antibodies that block autoantigen-mediated T-cell activation, leading to long-term T-cell downregulation and reduced cytokine production, including interleukin and interferon, which mitigates inflammation [7,8,17,18]. Although hIVIG has not been reported for the treatment of NNBMD in small animals, it has been used in several immune-mediated diseases in dogs, including immune-mediated hemolytic anemia, immune-mediated thrombocytopenia, Evans syndrome, pemphigus foliaceus, and myasthenia gravis [7,8,19,20]. Compared with cytotoxic chemotherapeutics or high-dose glucocorticoids, hIVIG is more readily available in clinical practice and is generally associated with fewer acute adverse effects. Based on these considerations and the documented use of IVIG in human bone marrow disorders, hIVIG was administered as an adjunctive immunomodulatory therapy in two dogs with refractory NNBMD, representing the first reported use of hIVIG for this indication.

The immunologic actions of hIVIG, including Fc-receptor blockade, modulation of cytokine synthesis, inhibition of complement activation, and Fas-ligand-mediated apoptosis, may suppress pathological immune responses targeting erythroid precursors in canine PIMA and MDS, providing a rationale for its therapeutic use in refractory NNBMD [7,8,17,18,21]. Immunoglobulins (Ig) also function as immunomodulators through interactions with autoreactive membrane receptors, such as Fc proteins on immune cells, and human Ig binds effectively to canine lymphocytes and monocytes [7,17,21]. Based on these mechanisms, hIVIG is hypothesized to downregulate phagocytosis of erythrocyte precursors by blocking macrophage Fc receptors and may induce immune regulation by reducing T-cell activation and proliferation in PIMA and MDS. Consequently, both cases demonstrated clinically meaningful hematologic stabilization following adjunctive hIVIG administration, characterized by maintenance of hematocrit above 20% and prolongation of transfusion intervals. However, these findings do not indicate complete hematologic remission. In particular, in case 2, reticulocyte production index values remained intermittently low after hIVIG administration, suggesting persistent ineffective erythropoiesis despite clinical stabilization. Moreover, given the concurrent or prior use of multiple immunosuppressive agents, a definitive causal relationship between hIVIG and the observed hematologic improvements cannot be established. Accordingly, the observed responses are best interpreted as supportive hematologic responses, consistent with their immunomodulatory mechanisms described in humans.

In humans, hIVIG dosage ranges from 0.4 g/kg monthly for low-dose replacement therapy to 2 g/kg administered over 3–5 consecutive days for high-dose immunomodulatory therapy, typically infused over 6–12 h [18]. Low-dose regimens are administered at fixed monthly intervals for patients with primary or secondary hypogammaglobulinemia to maintain adequate serum IgG concentrations, whereas high-dose regimens are used in acute immune-mediated or inflammatory conditions, including immune thrombocytopenia, autoimmune hemolytic anemia, Kawasaki disease, and other severe autoimmune disorders [18]. In contrast, veterinary guidelines for hIVIG dosing, infusion intervals, or treatment frequency have not been established. Reported canine doses range from 0.5 to 2.2 g/kg with infusion times of 4–8 h, and most veterinary reports describe single-administration protocols rather than repeated courses [7,8]. Although various canine hIVIG doses have been reported, formal guidelines defining dosing intervals or reinfusion frequency remain absent in veterinary medicine, necessitating individualized, response-driven treatment decisions in clinical practice [7,8]. In human medicine, however, IVIG is commonly administered in repeated courses, with dosing intervals determined by clinical response rather than fixed schedules [18,22,23,24]. For example, patients with chronic inflammatory demyelinating polyneuropathy typically receive maintenance infusions every 3–4 weeks, whereas those with immune thrombocytopenia or Kawasaki disease often receive repeated courses only when platelet counts decline or clinical relapse occurs [22,23,24]. A similar response-driven approach has been reported in veterinary medicine [19]. A multi-dose canine myasthenia gravis series reported repeated hIVIG infusions administered at variable intervals, with each dose administered in response to clinical relapse or inadequate treatment response [19]. Therefore, in the present cases, a response-driven dosing strategy was adopted. Repeat hIVIG administrations were determined based on serial hematologic monitoring, including declining hematocrit levels, persistently low RPI, and recurrence of clinical signs of anemia, rather than fixed dosing intervals. Specifically, repeat dosing was considered when hematocrit declined to levels approaching or below 20%, RPI remained below 1.0, and/or clinical signs of anemia recurred during follow-up. This approach was selected due to the absence of established veterinary dosing guidelines and is consistent with IVIG treatment strategies used in human immune-mediated bone marrow disorders.

Because hIVIG is a xenogeneic human plasma–derived product, dogs may develop hypersensitivity reactions, particularly with repeated exposure [25]. However, anti-human immunoglobulin antibody formation was not evaluated in the present cases, and therefore, the degree of immunogenicity associated with repeated hIVIG administration could not be assessed. A canine myasthenia gravis case that received multiple hIVIG infusions developed acute hypotension, collapse, and facial edema during the fourth administration, indicating that severe anaphylaxis may occur even when earlier doses were well tolerated [19]. In contrast, a recent prospective study of 12 dogs with presumed immune-mediated thrombocytopenia reported no infusion-related adverse events, and no acute reactions have been documented in this population, suggesting that clinically significant hypersensitivity to hIVIG is uncommon in dogs [26].

In the present report, to minimize the potential risk of adverse reactions, the lowest dose commonly reported in both human and veterinary literature (0.5 g/kg) was selected and infused slowly over 6 h [7,8]. This dose was considered appropriate for repeated administration to reduce the risk of adverse reactions associated with xenogeneic immunoglobulin exposure. Premedication with antihistamines and corticosteroids, along with slow infusion rates, can mitigate infusion-related adverse reactions [27]. Antihistamines reduce mast cell-mediated histamine release that contributes to urticaria, flushing, and vomiting, whereas corticosteroids suppress cytokine amplification and late-phase inflammatory responses [7]. Gradually increasing the infusion rate further limits complement activation and minimizes abrupt exposure to IgG aggregates, recognized triggers for hypersensitivity [27]. Both dogs in the present study received chlorpheniramine as premedication, and no infusion-related adverse effects occurred despite repeated dosing. However, antihistamine premedication alone cannot fully prevent hypersensitivity, as repeated administration of xenogeneic immunoglobulin may induce anti-human IgG antibody formation or immune-complex reactions. Continuous monitoring of cardiovascular and respiratory parameters is therefore essential throughout the infusion and for several hours afterward. This highlights the importance of careful clinical monitoring, particularly during repeated hIVIG administrations, to promptly detect delayed hypersensitivity or immune-mediated adverse reactions.

This case report has several limitations. First, the therapeutic effects of hIVIG were evaluated in only two dogs, and therefore, the findings cannot be generalized to the broader NNBMD population. Moreover, although maintenance of hematocrit above 20% and prolongation of transfusion intervals are clinically meaningful outcomes, these findings do not represent complete hematologic remission and are more appropriately characterized as partial or supportive responses rather than curative effects. Importantly, the observed clinical and hematologic improvements cannot be considered definitive evidence of therapeutic efficacy, as causality cannot be established from a two-case report with non-standardized treatment protocols. Second, bone marrow evaluation was limited to cytologic examination, and bone marrow core biopsy was not performed in either case, which restricts comprehensive assessment of marrow architecture, particularly in the diagnosis of myelodysplastic syndrome. In addition, assessment of bone marrow iron stores was not performed. However, iron deficiency was considered unlikely, as the dog had been receiving ongoing iron supplementation prior to referral, which would have limited the interpretive value of iron staining at the time of evaluation. Although serum cobalamin and folate concentrations were within reference intervals, functional deficiencies at the cellular level cannot be completely excluded, as markers such as homocysteine or methylmalonic acid were not evaluated [28]. This represents an additional limitation in the assessment of contributory causes of ineffective erythropoiesis. Third, a standardized hIVIG dosing protocol for dogs has not been established; therefore, dosing schedules in the present cases were empirical and based on clinical and hematologic response rather than predefined criteria. Fourth, anti-human immunoglobulin antibody formation was not assessed, preventing evaluation of immunogenicity and long-term safety associated with repeated hIVIG administration. Finally, long-term outcomes were not uniformly monitored in both cases, indicating the need for larger, prospective studies to validate the efficacy, safety, and clinical applicability of hIVIG in canine NNBMD.

## 4. Conclusions

This case report presents the first clinical observations of hIVIG administration in two dogs with refractory non-regenerative anemia associated with NNBMD, including PIMA and MDS. These findings indicate that hIVIG may represent an adjunctive immunomodulatory approach in dogs unresponsive to conventional immunosuppressive therapy. Further large-scale, controlled clinical studies are warranted to clarify the efficacy, safety, optimal use, and cost-effectiveness of hIVIG in veterinary patients.

## Figures and Tables

**Table 1:** Patient characteristics and regenerative indicators pre- and post-hIVIG in two dogs. Signalment, diagnosis, and hematologic parameters of two dogs with non-neoplastic bone marrow disorders (PIMA and MDS) before and after treatment with human intravenous immunoglobulin (hIVIG). Values are presented as means with ranges. Transfusion refers to the average duration of hematocrit maintenance above 20%.

		Case 1	Case 2
Signalment	Breed	Maltese	Dachshund
	Sex	Female (spayed)	Female (intact)
	Age (y)	10	7
	Weight (kg)	3.2	9.3
Diagnosis		PIMA	MDS
Duration of immunosuppressive therapy (months)		12	20
Number of transfusions prior to admission (wks)		4 (51)	8 (92)
Mean HCT % (range)	Pre-hIVIG	17.7 (15.6–20.2)	15.9 (10.8–20.9)
	Post-hIVIG	24.5 (21.4–33.4)	31.4 (25.3–36.4)
Mean RPI (range)	Pre-hIVIG	0.42 (0–0.69)	0.27 (0.12–0.40)
	Post-hIVIG	3.81 (0.14–5.00)	1.34 (0.03–2.10)
Mean reticulocyte count K/µL (range)	Pre-hIVIG	58.1 (18.7–90.1)	49.2 (26.7–70.1)
	Post-hIVIG	81.0 (36.4–145.7)	157.5 (66–231.8)
Mean transfusions term (wks)	Pre-hIVIG	10.3	11.6
	Post-hIVIG	20.9	31.7

PIMA, precursor targeted immune-mediated anemia; MDS, myelodysplastic syndromes; HCT, hematocrit; hIVIG, human intravenous immunoglobulin; RPI, reticulocyte production index.

## Data Availability

The original contributions presented in this study are included in the article/Supplementary Materials. Further inquiries can be directed to the corresponding author.

## Supplementary Materials

The following supporting information can be downloaded at https://www.mdpi.com/article/10.3390/vetsci13020145/s1. Table S1: Immunosuppressive treatment history prior to presentation at our institution. Table S2: Raw hematological data for case 1. Table S3: Raw hematological data for case 2.

## Abbreviations

CRI	Constant rate infusion
CRP	C-reactive protein
HCT	Hematocrit
hIVIG	Human intravenous immunoglobulin
MDS	Myelodysplastic syndrome
NNBMD	Non-neoplastic bone marrow disorders
NRIMA	Non-regenerative immune-mediated anemia
PIMA	Precursor targeted immune-mediated anemia
RPI	Reticulocyte production index
